# Beyond the Ordinary: An Atypical Guillain-Barré Syndrome Case With Unique Characteristics

**DOI:** 10.7759/cureus.44008

**Published:** 2023-08-23

**Authors:** Victor D Acuña-Rocha, Luis A González-Torres, Claudia E Gómez-Hernández, Ramon A Martínez-Scweinfurth

**Affiliations:** 1 Department of Internal Medicine, Hospital Universitario Dr. José Eleuterio González, Universidad Autónoma de Nuevo León, Monterrey, MEX; 2 Department of Neurology, Hospital Universitario Dr. José Eleuterio González, Universidad Autónoma de Nuevo León, Monterrey, MEX; 3 Department of Critical Care Medicine, Universitario Dr. José Eleuterio González, Universidad Autónoma de Nuevo León, Monterrey, MEX

**Keywords:** plasmapheresis, motor weakness, polyneuropathy, pharyngeal-cervical-brachial, guillain-barré syndrome

## Abstract

We present a patient with pharyngeal-cervical-brachial Guillain-Barré syndrome (PCB-GBS) that progressed to a severe state followed by a quick recovery after treatment. This unique clinical course has not been documented previously and provides a potentially invaluable description of a novel GBS variant. A 42-year-old man arrived at the emergency department with a 24-hour history of dysphagia, weakness in his right arm, and bilateral shoulder weakness. Nerve conduction velocity testing revealed bilateral sensory and motor polyneuropathy, leading to the diagnosis of GBS with the PCB variant. Timely diagnosis and plasmapheresis treatment contributed to a complete recovery of muscle strength and reflexes. In cases resembling ours, it is imperative to contemplate the existence of rare Guillain-Barré variants. This case underscores the necessity of recognizing and addressing rare Guillain-Barré variants in clinical settings with similar presentations.

## Introduction

Guillain-Barré syndrome (GBS) is an aberrant autoimmune response preceded by infections. Progressive limb weakness develops approximately two weeks after the autoimmune stimulation and peaks by the fourth week [1,2]. *Campylobacter jejuni* is the most frequent causative agent for GBS development; other pathogens, such as Cytomegalovirus, influenza and Zika virus, chikungunya virus, and *Mycoplasma pneumoniae*, have been reported in the literature; vaccines (e.g., H1N1 influenza vaccine) are other examples of reported causes [3-5]. The association between GBS and *C. jejuni* infection and a higher incidence of antibodies against GM1 and GD1a gangliosides relate to severe disease with pure motor axonal affection [6,7]. The findings of a prospective study of more than 250 patients with GBS showed that Miller-Fisher syndrome (MFS) (5%) was the most frequent GBS variant* *and pharyngeal-cervical-brachial (PCB) (3%) the second [8]. The PCB variant of GBS develops a rapidly progressive oropharyngeal and cervicobrachial weakness associated with areflexia in the upper limbs and acute motor axonal neuropathy (AMAN) without acute inflammatory demyelinating polyneuropathy (AIDP) [9]. We present a distinctive case of GBS with an initial clinical course consistent with PCB-GBS that subsequently developed into a severe generalized presentation inconsistent with the previous state, resulting in later ICU admission and successful management.

## Case presentation

A 42-year-old male patient, devoid of any preexisting chronic conditions, substance usage, family disease history, and prior administration of an unspecified SARS-CoV-2 vaccine, arrived at the emergency department. He reported a 24-hour history of dysphagia, along with weakness in the right arm and both shoulders. During the clinical interview, he mentioned experiencing diarrhea 10 days before admission, which resolved spontaneously after one day. Initially, his vital signs included a temperature of 36.3 °C, blood pressure of 120/70 mmHg, and a heart rate of 82 beats per minute (bpm). On physical examination, the Lovett scale indicated an upper limb motor strength of 3/5 with no sensory, reflex, or additional pyramidal features affected. Lower limb function was intact, and there were no signs of ataxia or altered consciousness. Our initial suspicion was a GBS with a PCB variant.

**Figure 1:**
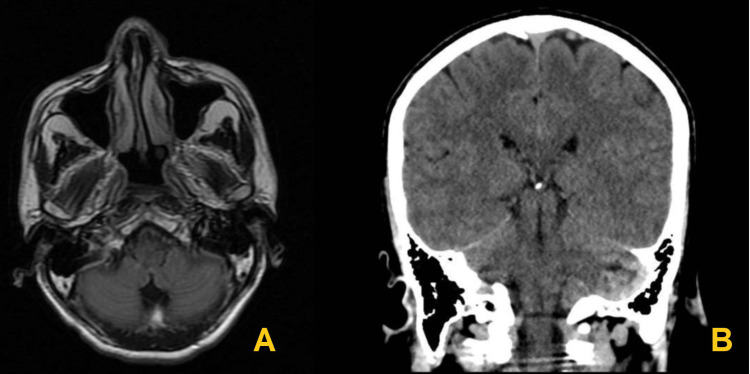
Head imaging. (A) MRI of the head in T2 FLAIR shows the output of cranial nerves IX and X without any alterations. (B) A simple tomography of the skull in the sagittal section, without structural alterations that compromise the anatomy of the head or trunk. MRI, magnetic resonance imaging; FLAIR, fluid-attenuated inversion recovery

A brain CT scan and MRI yielded unremarkable results (Figure 1). The lumbar puncture test demonstrated a protein level of 33 g/L, which after five days increased to 40 g/L. The otorhinolaryngology service conducted a nasendoscopy, revealing right cord paresis and a negative cough reflex. Given the patient's vulnerable airway and breathing difficulties, we quickly performed orotracheal intubation and transferred him to the intensive care unit.

**Figure 2:**
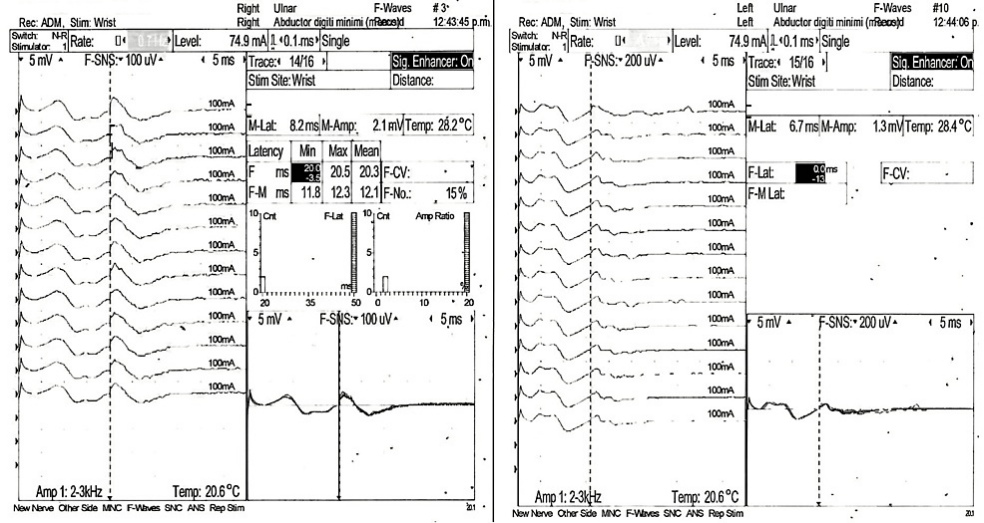
F-wave motor nerve conduction study of ulnar nerve at carpal level. Stimulation and response are recorded in the abductor digiti minimi muscle. The figure illustrates the bilateral absence of F-wave responses, indicative of impaired motor nerve conduction.

**Figure 3:**
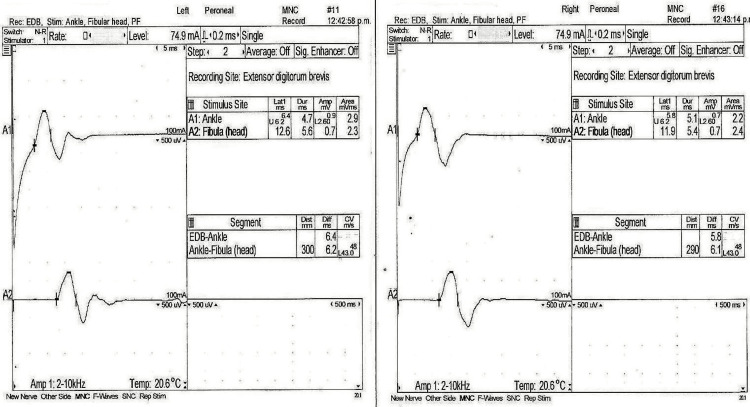
Motor nerve conduction study of the peroneal nerve. The left peroneal nerve shows diminished Amp and CV, with an Amp of 0.9 mV, DML of 6.4 ms, and CV of 48 m/s. The right peroneal nerve shows diminished Amp and CV, with an amplitude of 0.7 mV, DML of 5.8 ms, and CV of 48 m/s. Both studies suggest axonal involvement. CV, conduction velocity, Amp, amplitude; DML, distal motor latency

**Figure 4:**
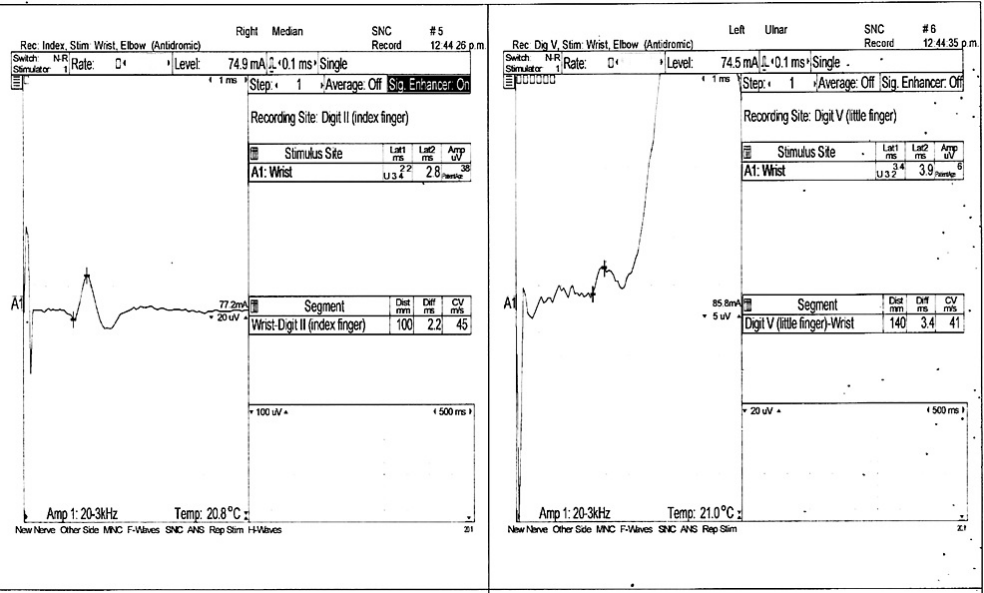
Sensory nerve conduction studies of the right median and left ulnar nerves. The right median nerve exhibits reduced CV and DML, yielding values of 45 m/s and 2.8 ms, respectively, with a normal Amp of 38 microV. The left ulnar nerve displays decreased CV and Amp, yielding values of 41 m/s and 6 microV, respectively, while maintaining a normal DL of 3.9 ms. CV, conduction velocity; Amp, amplitude; DML, distal motor latency

**Figure 5:**
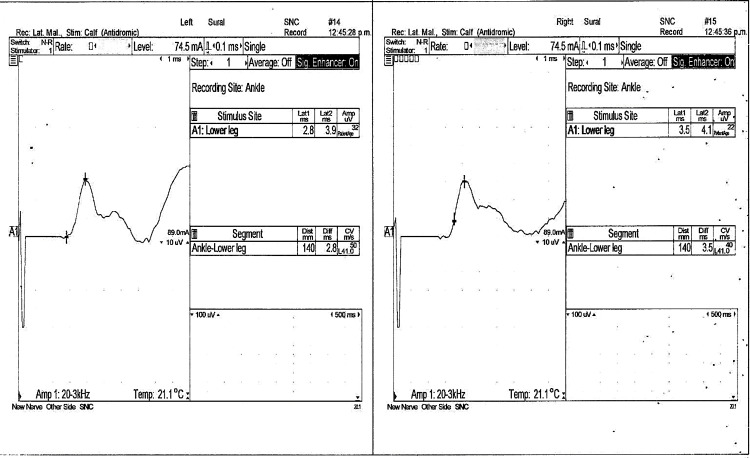
Sensory nerve conduction study in bilateral sural nerves. The right sural nerve exhibits normal Amp and DML, yielding values of 22 microV and 4.1 ms, respectively, and an altered CV of 40 m/s. On the other hand, the left sural nerve exhibits normal results, encompassing an Amp of 32 microV, DML of 3.9 ms, and CV of 50 m/s. CV, conduction velocity; Amp, amplitude; DML, distal motor latency

A nerve conduction velocity test of the arms and legs revealed a bilateral motor axonal and demyelinating sensory polyneuropathy (Figures 2-5). Additionally, we conducted antibody testing against the receptor of acetylcholine and muscle-specific kinase (MuSK), both of which yielded negative results. Serum ganglioside antibody GM1 testing resulted in a low titer positive result of <1:800. Notably, the patient developed areflexia within 24 hours after intubation.

Due to limited resources, we chose a five-day plasmapheresis treatment over immunoglobulin therapy for the patient. The patient's gag and cough reflex recovered after three days of treatment, enabling successful extubation. By the conclusion of the fifth day, following the completion of the plasmapheresis treatment, the patient had achieved motor recovery. Subsequently, the patient was discharged and scheduled for a follow-up appointment one month later, during which he reported a full and continued recuperation.

## Discussion

The strength of this case report lies in the remarkable distinctiveness of the clinical presentation associated with PCB-GBS, the unconventional trajectory of the clinical course, and the rapid recovery observed. These findings offer helpful insights for physicians dealing with similar clinical situations. The primary limitation of this study resides in the economic and availability challenges associated with conducting a comprehensive array of antibody tests when encountering GBS cases.

GBS variations are classified depending on the presenting clinical features in Table 1 [8,10,11]. PCB variant is considered a localized variation of GBS and has been increasingly reported [12]. Wakerley and Yuki proposed criteria for PCB diagnosis [9].

**Table 1:** Guillain-Barré clinical variants along with descriptions. .

Guillain-Barré variants	Clinical features
Miller Fisher syndrome	Ophthalmoplegia, ataxia, and areflexia
Bickerstaff brainstem encephalitis	Ophthalmoplegia, ataxia, areflexia, pyramidal tract signs, and impaired consciousness
Pharyngeal-cervical-brachial	Pharyngeal, cervical, and brachial muscle weakness
Pure motor	Motor weakness without sensory signs
Paraparesis	Paresis restricted to the legs
Bilateral facial palsy with paresthesias	Bilateral facial weakness, paresthesias, and reduced reflexes
Pure sensory	Acute or subacute sensory neuropathy without other deficits

Ropper, who studied three patients with progressive upper limb, oropharynx, neck, and shoulder weakness, and sensory loss with lower limb sparing, was the first to describe PCB of GBS. This variant accounts for approximately 3% of GBS clinical presentations [12-14]. Overlapping with other GBS variants may occur and can be considered a continuous spectrum [15]. Misdiagnosis can be frequent, considering clinical features that resemble myasthenia gravis (MG), stroke, or botulism. Typical PCB clinical features involve rapidly progressive oropharyngeal and cervicobrachial weakness associated with upper limb affection, lower limb sparing, and AMAN.

The patient came to us with difficulty swallowing, no cough reflex, and a gradual weakening of their upper limbs. He did not experience fatigue or decreased muscle reflexes. At first, we believed the patient had the GBS-PCB variant due to his preserved consciousness and lower limb sparing with paresthesia. However, after conducting nerve conduction studies, we discovered lower limb sensory and motor impairments that did not match our initial clinical findings. The patient's condition rapidly worsened, and we observed both axonal and demyelinating nerve features, along with areflexia and additional lower limb sensory and motor impairments. This made the case unique and challenging to align with established GBS clinical variants.

MG, cerebral ischemia, tumors, and botulism were considered differential diagnoses during the initial presentation; the evolution of the clinical course alongside the different battery of executed tests excluded all of them.

The treatment for GBS involves either intravenous immunoglobulin or plasma exchange. Our patient responded quickly to five sessions of plasma exchange, regaining complete upper limb strength after the third session. According to the literature, severe cases of GBS treated with plasmapheresis typically take around eight days to see improvement in motor function. Administering four or six sessions of plasmapheresis did not show a significant difference [16]. Our patient had a quick recovery, with motor function improving by day three and full recovery by day 30, making it a rare case of severe GBS with fast recovery. It's worth noting that steroids are not effective in treating this syndrome.

## Conclusions

The initial reflex sparing, followed by generalized areflexia, the presence of a demyelination syndrome, positive GBS serologic tests, negative serologic testing for MG, and the absence of ischemic or lesion findings in radiologic studies, in addition to substantial recovery after treatment, led to the final diagnosis of an atypical form of GBS with initial PBC presentation. The subsequent deterioration showed inconsistent clinical findings compared to our initial diagnosis.

Reporting of atypical GBS variant clinical presentations plays a vital role in understanding this pathology due to its diverse clinical spectrum. While numerous variants and overlapping syndromes exist, the distinctive features observed in this case have not been documented in previous literature, thus offering new insights and stimulating discussions for further advancements in GBS and GBS-PCB variant classification and management. The newly observed clinical course raises questions about whether it corresponds to an overlap syndrome, an atypical presentation of typical GBS, or a unique variation of PCB. Since there is no report of such clinical features, this could stimulate a new discussion regarding GBS and its variant classification system, potentially providing valuable insights for other clinicians.
